# Vaccine Development, Its Implementation and Price Setting: A Historical Perspective with Proposed Ways to Move Forward

**DOI:** 10.3390/jmahp13040050

**Published:** 2025-10-02

**Authors:** Baudouin Standaert, Oleksandr Topachevskyi, Olivier Ethgen

**Affiliations:** 1 Department of Care and Ethics, University of Hasselt, B-3590 Diepenbeek, Belgium; 2 Digital Health Outcome, UA-02137 Kiev, Ukraine; oleksandr.topachevskyi@digitalho.com; 3 Department of Public Health, Epidemiology & Health Economics, University of Liège, B-4000 Liège, Belgium; o.ethgen@serfan.eu

**Keywords:** vaccination, development, public health, economics, price setting

## Abstract

Vaccination has resulted in substantial public health benefits for human populations worldwide since it was first introduced more than a century ago. This article presents an overview of the history of vaccine development, its implementation, and price setting, the latter mainly from a developed world perspective. It considers potential issues and challenges. Over time, vaccine development and production has evolved to a market-driven approach, conducted largely by private commercial entities. The complex processes of identifying potential vaccine targets and developing and producing vaccines at scale have now become more efficient. However, vaccine pricing is an emerging concern. The elements that maximize the overall health benefit of vaccination include high volume, high coverage, and rapid initial implementation to achieve the high coverage with the vaccine as quickly as possible. It therefore requires substantial initial investment. Consequently, the price set for the vaccine should be reasonable to avoid limiting the coverage given the available budget. Suboptimal coverage leads to suboptimal benefit if herd protection is not fully achieved. This may disappoint health authorities and may result in program discontinuation. Conventional cost-effectiveness analysis is therefore not ideally suited to vaccine price setting, as it is based on the concept of ‘more for more’, i.e., higher health gain achieved at a higher reimbursement cost that does not account for limited budgets. Constrained optimization (CO) combines value assessment with constrained budget allocation into one analysis method and may therefore be the better option for vaccine pricing.

## 1. Introduction

Vaccination or immunisation was initiated with the aim of achieving better control of infectious diseases that were a threat to humans, covering endemic and/or epi-(pan)demic infections [1]. Vaccines are also widely used in the animal world as veterinary vaccines, with the same purpose of counteracting animal infections such as rabies, avian flu, or West-Nile virus [2]. Extended vaccine implementation can sometimes eliminate an infectious disease in humans within specific geographical areas including yellow fever, poliomyelitis, rubella, measles, or diphtheria [3]. Occasionally, vaccination can even lead to disease eradication, complete annihilation of the infection at global level, when there are no other infection reservoirs. This was first achieved for smallpox some 45 years ago and is now being approached for polio [4,5]. Disease elimination/eradication remains an important strategic goal of vaccination as a component of public health policy, aiming to reach quality health for the global population [6]. The World Health Organization (WHO) is helping to coordinate efforts to eliminate infectious diseases such as tuberculosis, malaria, rabies, or human papillomavirus (HPV) [7]. However, opposing views on vaccination policies are currently expressed, such as vaccine hesitancy [8,9]. This is partly a result of the success of vaccination [10]. Many vaccine-preventable infectious diseases have become rare over time as a result of vaccination programs, especially pediatric infections such as rubella, diphtheria, measles, varicella, polio, or mumps [11]. This may lead to questioning the need to continue vaccination, especially if there are vaccine safety concerns. In addition to vaccine hesitancy, other features are developing that may also jeopardize successful vaccination programs [12]. These will be the focus of this review, considering a historical perspective on the development of the current situation and then presenting prospects. Vaccine hesitancy will not be discussed here, as the problem is well-known, and there is already an extensive literature available and many working groups considering how it might be counteracted [13,14]. The present article will focus on less visible issues, including: the processes of vaccine conception, development, and production; the evolution of public health initiatives; and the price setting and marketing of preventative vaccines. Finally, the article will consider possibilities to help optimize the impact of vaccines on sustaining and enhancing population health at the global level.

## 2. Vaccine Development and Production

Vaccines were first introduced and generally used more than a century ago, with an initial focus on infants and children because infections were, and still are, most aggressive in that age group [15]. The innate immune response at birth against infections is too weak to produce an immediate and adequate immune reaction, and vaccination helps to overcome the suboptimal infant immune response. The science of vaccine development has evolved tremendously in the two centuries since the start of the first immunization methods, such as variolation against smallpox or injection of serum from suffering animals against rabies (Table 1) [16,17]. At that time, the mechanism of infection and immunization was unknown, and this first generation of vaccines was crude in preparing the antigen composition for the vaccine, which was selected through isolation and inactivation processes. 

The second generation of vaccines used cell-mediated cultures with advances in biotechnology to obtain higher quality and greater yields of antigens through recombinant DNA technology [18]. Once refined and well-controlled, this approach allowed for the mass production of vaccines that were both affordable and rapidly delivered. This period saw the successful production of vaccines against many infections, including typhus, cholera, plague, pertussis, tetanus, yellow fever, influenza, invasive pneumococcal disease, polio, measles, mumps, and rubella [19]. These vaccines came onto the market around the 1960–1970s, with little concern about purity and selection of the perfect antigen. The infections were highly prevalent in the population with easy spread and contagion, and the antigens associated with the pathogens causing the infections were rapidly and easily identified and used to create live-attenuated, inactivated, or toxoid-focused vaccines [15]. The next evolution in vaccine development employed more precise antigen selection to develop vaccines for use across a range of age groups (children and ageing adults) [20]. These techniques developed in the 1980s included chemical activation of conjugated vaccines with poly- and oligosaccharides such as glycoproteins, or structural adjustment of the antigen to obtain a better host immune response [21].

The next stage of vaccine development was more complex, because different antibodies and cellular immunity were required against antigens of pathogens with multiple strains, such as rotavirus, the Streptococcus pneumoniae bacterium, or HPV. The heterogeneity of the immune response therefore had to be increased. The critical feature of the antigens to be selected for a vaccine was the ability to activate neutralizing antibodies by the recipient [22]. These specific antibodies blocked the effect of the infectious agent causing illness and/or stopping its reproduction for disease spread. The identification of suitable antigens by evaluating neutralizing antibodies is now termed “reverse vaccinology” [23]. This refers to the use of genomic information to identify vaccine antigens that can induce a specific immune response through humoral or cell-mediated activities, before progressing into a vaccine development plan. It was sometimes very challenging to find the DNA/RNA genome codes, as lab-investigation and computer search often identified a very large number of different antigen candidates. However, the process underwent tremendous evolution during the past years thanks to dynamic computer programs, 3-D technology, and artificial intelligence (AI). At present, timelines for identifying suitable new vaccine antigen candidates are measured in weeks, in contrast to months or years in the past. This expedited process was recently tested with the response to the latest avian influenza virus infection (H7N9), which appeared in China, locally observed in humans in 2013 [24]. Through online databases that stock different virus genome templates, it was possible to identify a vaccine candidate in a record time of one week, although it still took years to test the candidate vaccine in clinical trials [25]. Other evolutions that have also enhanced vaccine effects in humans include the use of adjuvants, which are small molecules or materials incorporated in the vaccine composition to enhance the immune response [26]. Many developments occurred over time in developing different types of adjuvants, as no single adjuvant is key to all vaccines but different adjuvants are needed for different vaccines [19].

Quality assurance remains a key issue for the development of any new medicinal product (also discussed in the next section). Rigorous evaluation processes must also be followed for vaccines, adhering to established phases of clinical trials, numbered from Phase 1 to 5 [27]. Each trial phase requires time, often extended over one year, and reducing this timeline necessitates the implementation of parallel instead of sequential activities. This approach was effectively employed during the development of the COVID-19 vaccines, resulting in time savings of 2 to 3 years in this quality evaluation process [28,29].

In summary, the whole vaccine initiation and development process became significantly more efficient over the past 2 to 3 decades, aided by new robotic automation programs and other technological innovations. Consequently, the initiation and quality evaluation of new vaccines can now be accomplished more rapidly at a lower cost [30]. There is, however, an important issue with this evolution of information, knowledge and communication worldwide. The democratization process has enabled numerous groups across the world to initiate research on vaccine development but often working in silos. During the COVID-19 vaccine development period, over 150 research centers worldwide were reported to be involved in active research into a new vaccine [31]. Unfortunately, success leading to a commercial vaccine was achieved primarily by a few well-equipped groups with superior resources, extensive experience, and innovative strategies, which were not available to all research groups. Therefore, this experience raises questions about resource allocation of high investments in projects that ultimately did not prove successful. Moving forward, it will be important to support strategies that ensure that high funding is preferentially channeled into projects with a high probability of achieving successful end results, by fostering collaboration to enhance technical and cost efficiency. New international initiatives, such as the Coalition for Epidemic Preparedness Innovations (CEPI), are emerging, aiming to optimize a more efficient global vaccine development landscape against new infectious diseases that could develop into pandemics [32]. 

## 3. Development of Public Health Initiatives and Vaccine Implementation Programs

The previous section presented an overview of vaccine initiation and development over time, looking forward to a future with better international collaboration. This section presents a summary of the development of vaccination implementation programs in the past and present. 

By the end of the 19th century, many public health programs at country level were initiated to stimulate and improve local/home hygiene conditions, to ensure access to clean water, fresh air, nutritious food, and to control infections, especially at that time of tuberculosis (respiratory) and syphilis (a sexually transmitted disease) [33]. Once it was understood that germs were the cause of infections, they had to be detected, and transmission had to be prevented. Public health institutes (PHI) were established at various administrative levels, sometimes national level and sometimes at the provincial level, particularly in countries like Belgium, to oversee the implementation of public health strategies [34,35]. Considerably later (10 to 20 years later), shifts to treatment of infectious diseases became a reality after the discovery of antibiotics [36]. These early PHIs were in some countries, such as Belgium, The Netherlands, and the Nordic countries in Europe, instrumental in not only informing, educating, and promoting hygiene but also in producing the first generation of vaccines (see Table 1) [35,37]. These PHIs were crucial in manufacturing critical vaccines, such as those against typhoid fever which had a profound impact on the health condition of soldiers during World War I [38]. Initially, vaccines were locally developed that focused on preventing infections in children, who were most vulnerable to diseases like polio, which can lead to permanent paralysis of limbs and respiratory organs. However, not all vaccines were produced at that time by public organizations. Some private groups in Germany (Behringwerke), Italy (Sclavo Istituto), and the UK (Burroughs-Wellcome) took up the challenge to produce anti-toxins for commercial purposes, given the increased market in colonial settings outside their home countries [37].

In the mid-20th century, societal dynamics rapidly evolved, particularly in the wake of World War II. New treatments for various diseases were introduced, alongside the emergence of regulatory frameworks to ensure the quality, safety, and efficacy of these products. International quality standards for pharmaceuticals became necessary after several high-profile cases of adverse effects, such as the thalidomide tragedy, which highlighted the dangers of inadequate safety testing [39]. As discussed earlier, the process of assessing quality and safety of new products was often complex, time-consuming, and costly [27]. The process began in the 1950s and gradually evolved into more sophisticated programs as used today. New vaccines coming into the market also had to undergo these evaluations of strict product quality and safety norms [40]. However, it was more difficult to perform the studies for vaccines focusing on prevention than for drugs directed for the treatment of illness, especially if the criteria for evaluating the vaccines were based on clinical effect and not on transmission. Clinical effects have a much lower rate of manifestation than transmission, because not every infected person displays symptoms. Consequently, for vaccines, large groups of individuals must be tested and followed in clinical trials with long periods of observation. As an example, a rotavirus vaccine was tested on around 4000 people in a Phase 3 trial in Europe during 2 years of observation [41]. There was therefore a pressure to employ multi-country approaches in order to rapidly assemble and test adequate numbers of people at the same time. As a result, it was difficult to rely on local PHIs doing those evaluations in developing and testing new vaccines, as they had limited resources available and were missing international collaboration and networking, although there was value in having public institutes conceiving the potential for new research and development, as discussed by Stuart Blume [42]. Large pharmaceutical companies were better equipped and positioned to navigate cross-border regulatory landscapes, having established extensive international networks. These companies also recognized the profitability of vaccines, as they potentially secured a steady influx of demand/income when systematically vaccinating specific populations such as newborns. Therefore, the need for international and not only local development puts PHIs in a weak position to continue new vaccine development programs. Private companies also began to dominate production and distribution of vaccines, while the institutes pivoted into other roles such as laboratory research and epidemiological studies, which were also increasingly handled by universities or private entities. Moreover, the administration and distribution of vaccines shifted from the institutes to medical providers such as general practitioners and pharmacies, which became a new source of income in the healthcare sector. 

The PHIs, therefore, increasingly focused their work on marginalized populations, such as the elderly, immigrants, the socioeconomically disadvantaged, and/or prisoners. They also redirected their efforts towards environmental health and the investigation of biological diversity [35,43]. In addition, with the introduction of many different vaccines, infections were becoming rare, and the public health focus therefore changed to problems that were no longer infection-related but focused on non-communicable diseases. A list of the most prevalent public health issues in the Western world in the pre-COVID period published in 2016 does not include any infection-related concern. The focus was rather on smoking, alcohol abuse, drugs, obesity, physical activity, social isolation, unhealth food, dementia, and the like [33]. A more recent inventory, post-COVID-19 pandemic briefly placed infectious diseases back on the radar [44]. 

During the pandemic, private and multinational organizations, rather than the original PHIs, played a more important role in managing the crisis in some countries. However, the role of the national institutes of health in a country remained prominently present for monitoring the disease, projecting infection simulations, and informing educational guidelines on how to correctly behave to reduce the infection risk [45,46].

The transition of public health responsibilities from public to private entities represents a significant shift in the healthcare landscape regarding impact and payment, and it is also the cause of another important emerging issue. Increasing demand for operational efficiency, combined with the growing complexity of healthcare needs and market-driven dynamics, led to the privatization of many healthcare tasks [47]. The end result was a situation in which public health priorities were often shaped by market forces rather than by public welfare [48]. This raises questions about the best way to address the evolving challenges in public health related to vaccination. For example, finding the optimal implementation strategies for new vaccines to maximize long-term success, or monitoring shifts in virus strains over time potentially making existing vaccines less effective [49]. There is a question over whether epidemiological research and public health interventions should primarily occur at national/country or at local level for maximum effectiveness [50]. With the advent of digital technologies and the growing availability of healthcare data, there is a potential to return to more localized public health models, where local concerns can be more directly addressed [51]. This move could potentially offer new opportunities for revitalizing local public health entities, enabling them to play a prominent role in health strategies and welfare, potentially in collaboration with broader efforts in an international network. This could be most helpful to address the growing concern of ageing people and society [52]. However, such a transition would require a clear vision, strategic planning, and a re-evaluation of the roles and functions of both public and private sector actors in the future of global public health development.

## 4. Price Setting of Vaccines

The shift from public to private organizations in the development, production and administration of vaccines raises the question of price setting for a new product when the market is essentially privately run. A rational approach to the pricing of new medical drugs coming onto the market emerged approximately fifty years ago in the United States [53]. It sought to establish a framework for determining acceptable prices of medical interventions, primarily based on the clinical benefits offered and the associated costs in achieving these benefits [54]. At its inception, this framework emphasized the definition of an outcome measure such as improved survival or reduced morbidity that could be linked to the new intervention, and at the same time aimed to define the incremental costs required to achieve those clinical benefits. However, these costs were not production or service costs, but reimbursement prices established by public authorities [55]. This viewpoint was selected because it was the perspective considered in public financing of healthcare, with a price analysis focusing on reimbursement paid by social security funds [56]. During the past 3 decades much research led to the development of new evaluation methods, now called health economics, to focus on acceptable price setting in healthcare. The healthcare environment differs from that in normal market situations where prices are set through offer and demand, which is termed as the ‘free market’ situation [57]. Although there is offer and demand in healthcare, if the rules of free market activity were to be applied, the healthcare market may spontaneously evolve towards distorted price settings of new products with a distorted reach of people most in need of the medical goods [58]. Thus, healthcare authorities had to intervene in the price setting of healthcare interventions, and this was done by requesting health economic submission dossiers by the applicant in order to obtain reimbursement of new medical products [59]. A health economic dossier must demonstrate the value of the new product through cost-effectiveness analysis (CEA) before the product will be reimbursed. Being cost-effective means that the price proposed for the new product must show good value for money, when compared with the existing disease management situation. ‘Good value for money’ is conventionally defined as the outcome of the CEA calculation being under the threshold for what is normally paid for one unit of extra clinical gain by society. This threshold value is country-specific, although a commonly used average in the Western world is a price of 50,000€ per Quality Adjusted Life Year (QALY) gained (QALY is a standard measure used for defining the clinical benefit in healthcare) [60]. By applying this CEA guideline, authorities aim to achieve equal access to the medical product for the population at an affordable price. However, the underlying assumption of CEA is ‘more for more’ - more clinical gain justifies a higher reimbursement price. This approach is not aligned with the way the rest of the healthcare budget is spent for purposes other than for the reimbursement of new medical drugs and devices. Payment for other medical activities, such as hospital care or other logistical care including home care, first-line care, pharmacies, physiotherapy, and the like, is generally fixed per activity and not linked to the achievement of an outcome measure. Moreover, the total budget available for healthcare is fixed per year with a politically agreed annual increase [61]. Conventional CEA evaluation may demonstrate that a new product offers sufficient additional clinical effect per unit of additional expenditure to be considered ‘good value for money’ according to the accepted threshold, but that does not account for how the additional expenditure could be accommodated within the fixed budget. The pharmaceutical industry tends to add additional projections, models, (indirect) costs and effects to demonstrate that the ‘clinical’ more-for-more price, while being cost-effective, could underestimate the economic value of the new product. However, this may be influenced by the assumptions used in the models and the analyses conducted to be shown to the authorities to support the desired reimbursement price at product launch. 

Vaccine price setting has generally followed the same economic evaluation rules as for treatment interventions using the same CEA approaches [62,63]. Meanwhile, national authorities have developed and organized advisory boards such as National Immunization Technical Advisory Groups (NITAGs), applying health technology assessment (HTA) tools to receive input into the immunization program to be implemented at country level and the price to be paid [64]. The currently best known NITAGs are the Advisory Committee on Immunisation Practices (ACIP) in the US, the Joint Committee on Vaccination and Immunisation (JCVI) in the UK, and the Standing Committee on Vaccination (STIKO) in Germany. They all have their own rules of evaluation, which sometimes move in the same direction, although not always because of local conditions. National authorities had to organize these specific committees because the focus of vaccines and vaccination is on public health and disease prevention, avoiding or reducing the risk of developing illness from a vaccine-preventable disease in a population at risk. This differs from the focus of treatment, where the aim is to improve through care and/or cure the individually impaired health condition of a sick person. With a preventive intervention, the individual receiving the intervention is in good health and not currently sick. If prevention works well, nothing should change at either the individual or the population level, because the vaccination blocks the spread of the infection and the individual and population do not develop the disease targeted by the vaccination program. However, the vaccine must be paid for, to achieve this apparent lack of change. And that is often difficult to sell and to promote [65].

Maximizing the public health benefit from vaccination depends on achieving high coverage levels to obtain the indirect effects of vaccination (herd protection) [66,67]. Achieving broad population coverage requires balancing the volume of doses administered with affordability [68]. This is an issue for vaccine price setting, because in this context, a high per-dose price may be counterproductive if the initial investment cost to achieve rapid widespread immunization puts too much strain on the available budget. While it is important to assess the broader societal benefits of vaccination—such as reduced long-term healthcare costs, avoided productivity losses, and improved population health—it may become problematic if these broader economic impacts are used to negotiate higher prices that then limit the coverage achievable within the available budget. The primary goal of a vaccination program should be to maximize the health benefits of vaccination by ensuring affordable access to the vaccine within short time windows for the total at-risk population. The right balance must be found between coverage and affordability to be taken into account in the vaccine price setting [69].

## 5. The Future

The preceding sections have given a broad overview of the historical development and current position of vaccine development, program implementation, and price setting. Aside from vaccine hesitancy, this section will consider factors that could potentially jeopardise the further development of vaccination successes in public health, including countering future unknown pandemic threats. There are currently several different economic forces influencing the vaccine market in different directions (see Table 2).

One force is the development of new vaccines, as obtaining the antigen codes of the infectious agent and making the information easily available to everyone that can now be achieved in a more efficient and timely process than ever before. This effort can be spread worldwide, potentially allowing new infection pandemics to be characterized in record time as needed. In a globalized world, pandemics can potentially spread worldwide, sometimes with local concentrations [71]. Given the limited resources and time to develop a response when new pandemics appear, it would be better to optimize the vaccine development process further. This remains a challenge, and an analogy could be drawn with climate change where solutions need to be found that offer benefits for politicians, producers, and the population. The role of vaccine research and development is too important to global public health to be left solely in the hands of large corporations whose primary objective is maximizing profit. Many infectious agents are more prevalent in developing countries, where the lack of resources leads to absent or significantly underestimated reports of infection incidence and prevalence. Such situations are associated with low commercial interest, due to the lack of potential profit, leading to low prioritization of vaccine research and development by commercial companies [72]. 

Over time, the ability of local public health organisations to coordinate, implement, and follow-up vaccination effects in the field has been lost. Most of this role has been taken over by private and/or national organisations, raising questions over the most efficient approach to fulfil all these different tasks. The switch from public institutes to private organisations for testing, producing, and disseminating new vaccines has resulted in faster development, increased knowledge, and greater focus. However, with regard to implementation, education and monitoring of vaccine impact, the optimum level of public versus private input can be debated. These tasks need an objective approach to ensure that correct evidence-based information is collected, guaranteed and disclosed, and this may be more credible if carried out by public bodies than commercial profit-driven organisations. 

Price setting of vaccines has evolved in two directions over time. One is through the process of tendering. Historically, vaccines have been exposed to tendering processes more than other medical drugs or device, because tender requests for vaccines were quite easy to set up. Consequently, price erosion may occur over time if the tender processes are too frequent and too many producers are competing (influenza vaccine price setting is an example) [73]. Some countries, such as the UK, have developed specialised processes and have a transparent approach to organising tenders without disclosing the price paid. However, if the tender price becomes too low it may reduce the number of producers, and this may create problems with insufficient vaccine production, as it has sometimes occurred with influenza vaccines available in the US [74].

The second approach historically used for vaccine price setting is the application of classical CEA. An emerging concern at present is that the large, private pharmaceutical companies developing and producing vaccines have a major focus on short-term profit generation for the company, applying the same logic of maximising profit used in the treatment area to public health vaccination programs without making any distinction [75]. However, if vaccine prevention becomes too costly, the high volume of coverage needed to achieve optimal health benefits from the vaccination program will not be reached [76]. Consequently, health authorities may be disappointed by the results obtained by suboptimal prevention programs, and the programs may therefore be discontinued. The problem is not a lack of vaccine efficacy, but that the payment per dose is too high to achieve rapid high vaccine coverage within the constraints of available healthcare budgets. This suggests that CEA can be helpful as an indication of the vaccine price defined by its value, but the coverage volume achieved is the major driver for a successful vaccination program. Maximum long-term profit may be achieved more readily through volume increase than through price increase. To achieve higher volume the producer needs to be the best and/or the first. For maximising revenue and profit the focus is on market share, cost constraint, and other logistical constraints, which may be better assessed using optimisation modelling rather than CEA [77,78]. Ultimately, as public funds are used to finance vaccination programs with the objective of preventing disease, vaccine price setting decisions should prioritize the public health goal of maximizing coverage and thus maximizing benefit, rather than focusing on short-term profitability of private companies. This requires balancing the costs of development, production and distribution with the imperative to optimize access and effect. Moreover, local authorities should reinforce economic evaluation guidelines to support price setting for new vaccines that are compatible with achieving the necessary extent and speed of coverage for a successful vaccination program. 

Although tender or CEA are the two conventional approaches for price setting of vaccines, both have weaknesses. Constrained optimization (CO) is better suited to vaccine price setting, as working within a fixed budget is the reality in most healthcare systems. In this approach, the available budget is pre-defined, and the analysis identifies the product or combination of products that achieves the maximum effectiveness and would then win the market. A submission dossier could present a list of additional, societal and economic gains that can be achieved with the vaccine, for example, using the cauliflower approach [79]. This additional societal gain can be used to argue for the value of the vaccination program, but it should not be used to increase the vaccine price to a level that may limit coverage to suboptimal levels given the constraint of the available budget. For vaccines and public health interventions producers should evaluate whether the potential long-term gain achieved by a strategy of sustained high coverage might exceed the short-term gain achieved by a high per-dose price but with a lower volume and/or the risk of program cancellation if results are sub-optimal. In addition, making the vaccine attractive through appropriate design and administration should be part of supporting higher uptake and coverage, but should not be used to obtain a higher price at the expense of coverage. 

An optimum scenario for development, implementation and price setting for future public health preventive vaccines should include sharing the best information on antigen selection, standardizing a maximally automated production process with worldwide reach, optimizing the mix of private and public organisations to inform, educate, and administer the vaccine with adequate follow-up. These are constraints that should be included in the constrained optimization (CO) calculation [77,80,81,82,83]. This CO approach allows an integrated analysis to maximize health outcomes, within the constrained conditions of available budget, logistic capabilities, response rate, and others. It identifies the best combination of different interventions that reach an optimal result while complying with the constraints. It is therefore more powerful than CEA, as it does not rely on the artificial construction of threshold payments, and it can take account of the reality of a fixed budget. The attraction of the CO method is that it can answer two important questions for a decision-maker seen from different perspectives, which are as follows: the budget needed to reach a specific clinical outcome (such as a 10% reduction in disease-specific mortality within a defined period of 5 years); or the maximum disease-specific mortality reduction that can be reached within 5 years, given a fixed budget. Using this approach, the vaccine price may be more or less fixed by the budget available to the authorities, but the product or combination of products that demonstrates the best clinical effect within the budget would be selected for the program. The process could be repeated at intervals as other alternatives become available. With the right balance between the work cost for the product (development, production, implementation and follow-up, allowing for a reasonable profit margin) and the health gain obtained at the population level, such an approach could create the incentives needed to encourage competition between private entities to develop and produce new vaccines. Creating such an international framework that optimizes the future development of new vaccines will provide essential public health benefits for all human beings worldwide.

## Figures and Tables

**Table 1:** Steps in vaccine development over time.

Generation	Steps	Since
1st	Inoculation (vaccination)	1796 (smallpox)
	Isolation & inactivation (empirical)	1870–1890 (cholera, anthrax, rabies)
2nd	Cell-mediated vaccine development	1950 (polio)
	Recombinant DNA	1980 (Hep B) 2006 (HPV)
	Conjugation (glycoproteins)	1990 (Hib, IPD)
3rd	Reverse vaccinology Structure-based antigen design	2000 (Men B) 2013 (RSV)
4th	Synthetic Biology	2014 (new Flu) 2020 (SARS-CoV-2)
	AI-supported vaccine candidates	2024

Hep B: Hepatitis B; HPV: Human Papillomavirus; Hib: Hemophilus Influenza B; IPD: invasive pneumococcal disease; Men B: Meningococcus B; RSV: Respiratory Syncytial Virus; SARS: Severe Acute Respiratory Syndrome; CoV: Corona Virus.

**Table 2:** Summary of the economic issues and proposed solutions to maintain successful vaccination programs.

Activity	Question	What
Vaccine initiation & development	Achievement	Genome composition of the pathogen communicated worldwide
	Issues	Too much silo research dissipating time, effort, money, and energy
		Lack of efficiency evaluation of allocated resources
	Solution	Limit numbers of research centres
		Coordinate the efforts between the centres to be more efficient
		Increase technical skills to control new pandemics under optimal time scenarios
Vaccine production, implementation and monitoring	Achievement	From public initiation to private endorsement of production
		Production implementation guarantees quality & efficiency
	Issues	Degrading public institutes to marginal roles
		Market-driven focus on vaccine production in competitive environment
		No focus on:
		- Optimal vaccine launch strategy
		- Monitoring shifts in pathogen strains
		- Evaluating changes in effectiveness through reduced immune response, new sources of infection, or limited herd effect
	Solution	Search for improved and maintained population well-being
		Develop adequate methods of local data collection
		Create responsible actions through local public health organisations
Price setting	Achievement	Cost-effectiveness analysis (CEA) in line with treatment assessment
		Tender processes
	Issues	Higher profit through higher pricing by including societal benefits
		Too frequent tenders causing price erosions
		This leads to fewer producers
		Fewer producers lead to vaccine shortages when most needed
	Solution	High volume, high coverage of the at-risk group within short time frames
		Price setting for attaining high coverage using constrained optimization (CO)
Vaccine hesitancy	Achievement	Making the inventory of the causes and spread of disinformation
	Issues	Too many stakeholders to communicate about risk-benefit assessment
	Solutions	Better communication in simple terms (infographics) to convince the 6P’s [70]

## Data Availability

No new data were created or analyzed in this study.
